# Influence of Image Acquisition on Radiation Dose and Image Quality: Full versus Narrow Phase Window Acquisition Using 320 MDCT

**DOI:** 10.1155/2013/731590

**Published:** 2013-12-23

**Authors:** Faisal Khosa, Atif Khan, Khurram Nasir, Waqas Shuaib, Matthew Budoff, Ron Blankstein, Melvin E. Clouse

**Affiliations:** ^1^Department of Radiology, Emory University Hospital Midtown, Atlanta, GA, USA; ^2^Department of Radiology, Beth Israel Deaconess Medical Center, Harvard University, Boston, MA, USA; ^3^Department of Cardiology, Yale University School of Medicine, New Haven, CT, USA; ^4^Department of Cardiology, Los Angeles Biomedical Research Institute, Torrance, CA, USA; ^5^Department of Radiology, Brigham and Women's Hospital Harvard Medical School, Boston, MA, USA

## Abstract

*Purpose*. To compare radiation dose and image quality using predefined narrow phase window versus complete phase window with dose modulation during R-R using 320-row MDCTA. *Methods*. 114 patients underwent coronary CTA study using 320-row MDCT scanner. 87 patients with mean age (61 + 13 years), mean BMI (29 + 6), and mean heart rate (HR) (58 + 7 bpm) were imaged at predefined 66–80% R-R interval and then reconstructed at 75% while 27 patients with mean age (63 + 16 years), mean BMI (28 + 5), and mean HR (57 + 7 bpm) were scanned throughout the complete R-R interval with tube current modulation. The effective dose (ED) was calculated from dose length product (DLP) and conversion *k* (0.014 mSv/mGy/cm). Image quality was assessed using a three-point ordinal scale (1 = excellent, 2 = good, and 3 = nondiagnostic). *Results*. Both groups were statistically similar to each other with reference of HR (*P* = 0.59), BMI (*P* = 0.17), and tube current mAs (*P* = 0.68). The median radiation dose was significantly higher in those scanned with complete R-R phase window versus narrow phase window (*P* < 0.0001). Independently of patient and scan parameters, increased phase window was associated with higher radiation dose (*P* < 0.001). Image quality was better among those scanned with narrow phase window versus complete phase window (*P* < 0.0001). *Conclusion*. Our study supports that good HR control and predefined narrow window acquisition result in lower radiation dose without compromising diagnostic image quality for coronary disease evaluation.

## 1. Introduction 

MDCTA has emerged as a robust technique for the assessment of coronary artery anatomy and disease with accuracy comparable to invasive coronary angiography [1–5]. Owing to a high negative predictive value, coronary MDCTA may be suitable as a primary screening tool for those symptomatic patients with low probability of coronary artery disease [1]. While specific clinical applications of MDCTA are still the subject of some debate, improvements in temporal and spatial resolutions have substantially broadened its potential use. Nevertheless, the enthusiasm for MDCTA has been tempered by concerns about the potentially high radiation dose received while undergoing coronary MDCTA and its attendant potential cancer risks [6, 7].

LaBounty et al. showed that decreasing the phase window was associated with substantial reduction of radiation dose with preserved image quality using 64-row scanner [8]. However, a prior study using the 320 MDCTA suggested that narrowing the phase window width also has the potential to reduce diagnostic accuracy [9]. The purpose of our study was to compare the radiation dose and image quality of data captured during the cardiac cycle centered at 66–80% of the R-R interval (narrow phase window) versus complete phase window during entire R-R interval with tube modulation (functional imaging with dose modulation) using 320 MDCTA.

## 2. Material and Methods

### 2.1. Study Population

This retrospective study was approved by our Institutional Review Board and informed consent was waived. The study population is comprised of consecutive series of one hundred and fourteen patients undergoing clinically indicated prospective ECG-triggered MDCTA (Table 1). Imaging modes were chosen based upon their respective referral reasons. (Group A) 87 patients (mean ages of 61 ± 13 years, 72% males, mean BMI of 29 ± 6 with mean HR of 58 ± 7 bpm) referred for coronary artery disease evaluation were imaged at 66–80% of R-R interval while (Group B) 27 patients (mean age of 63 ± 17, 52% males, mean BMI of 28 ± 5 and mean HR of 58 ± 7 bpm) referred to determine coronary artery disease plus functional status of heart were imaged throughout R-R interval with tube current modulation. Inclusion criteria for coronary MDCTA were atypical chest pain, suspected CAD, pathological ECG results or equivocal stress test, dyspnea, and cardiac risk factors. Clinical exclusion criteria for exam were nonsinus rhythm, severe allergy to iodine-containing contrast material, history of renal disease (calculated from creatinine levels > 1.7 mg/dL), pregnancy, hemodynamic instability, and severe respiratory or cardiac failure. Patients' DICOM images were reconstructed at Vitrea workstation to review segmental image quality.

### 2.2. CT Angiography

All MDCTA examinations were supervised by a cardiovascular imaging fellowship trained radiologist with seven years of clinical experience. Patients were connected to ECG leads placed in standard position to enable CT synchronization with ECG. Beta blocking medication (oral or intravenous metoprolol) was administered to achieve heart rates <65 bpm unless contraindicated to avoid motion artifacts. Sublingual nitroglycerine (0.4 mg) was administered to dilate coronaries for coronary vessel evaluation. There were no patients with contraindication to beta blockade or nitroglycerine in our study.

All MDCTA scans were performed with a 320-row scanner (Aquilion ONE, Toshiba, Tokyo, Japan). The starting point of the volume scan and coverage area was cranio-caudally from one centimeter below the tracheal bifurcation to the diaphragm. Prior to the examination, all patients were instructed on quiet breathing and breathe holding in order to minimize artifacts during scanning. The Optiray 350 (Ioversol injection 74%; 70−100 mL) was injected at a rate of 5 mL/second followed by 50 mL of normal saline at 5 mL/sec. The scan parameters were determined on basis of patients BMI. All patients in both group were scanned using 120 kVp, 400 mA, gantry rotation 350 msec, 320-row (0.5 mm detectors), and 160 mm volume scan length (Table 2).

### 2.3. Scanning Modes

**Figure 1:**
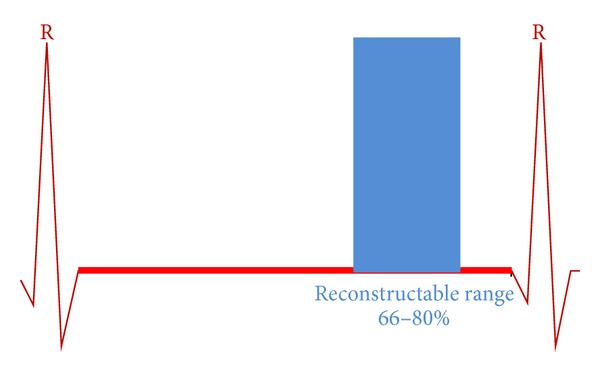
Narrow phase window (66–80) showing limited tube current exposure, 72 × 29 mm (300 × 300 DPI).

**Figure 2:**
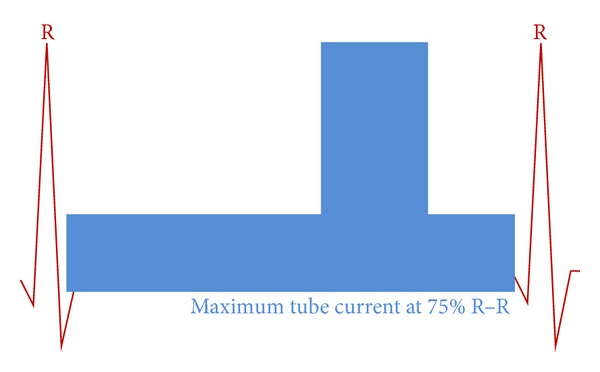
Complete phase window with tube modulation, 76 × 39 mm (300 × 300 DPI).

*(A) Prospective Narrow Phase Window (66–80%).* Figure 1 depicts the R-R interval of the cardiac cycle in seconds (abscissa) and the height of the shaded areas represents the tube current (mA). For the volume scan as depicted in the figure, the applied tube current is at a constant preset (mA value) to 66–80% of the R-R interval. This scheme will result in a lower dose to the patient compared to the other modified tube current scheme shown in Figure 2.

*(B) Complete Phase Window Acquisition with Dose Modulation.* Figure 2 depicts that tube current is modulated and applied throughout the R-R interval for the cardiac scanning (cardiac perfusion and functional studies). The tube current is increased between 66% and 80% of the R-R interval. However images can be reconstructed throughout the cardiac cycle for analysis.

### 2.4. Assessment of Image Quality

Image analysis was performed by two readers who were level-3-certified competent by the Society of Computed Cardiac Tomography. The images were reviewed at the Vitrea workstation V4 FX version. The readers were able to scroll through axial images and interactively perform multiplanar reconstructions and maximum intensity projections, as well as curved multiplanar reformats for both data sets. After blinding the examinations, the image quality of the coronary vessels was assessed subjectively by the two observers using the 16-segment American Heart Association model [10]. All patients were analyzed in a randomized manner and all segments were evaluated using an ordinal scale from 1 to 3 (1, excellent; 2, good; and 3, nondiagnostic). Segments assigned to a score of 3 were not evaluated because of marked motion artifacts, structural discontinuity, image noise, related blurring, or poor vessel opacification.

### 2.5. Calculation of Radiation Dose Estimates

The effective dose (ED) was selected as the best measure to assess and compare radiation dose exposure. The ED shows the nonuniform radiation absorption of partial body exposure relative to whole body radiation. For ED calculation, the dose length product (DLP) was multiplied with a conversion factor *k* (0.014 mSv/mGy/cm). The DLP was recorded from the scanner console (see [11–13]):

(1) effective  dose  (ED)=dose  length  product  (DLP)×k.

### 2.6. Statistical Analysis

Median radiation doses were reported. Median radiation dose difference was compared by using Kruskal-Wallis equality of population rank test. Further regression analysis was performed for median radiation dose for both groups adjusted for other variables (age, gender, BMI, heart rate, tube voltage, and tube current). A *P*  value < 0.05 was considered statistically significant. Ordinal scale was used to interpret the image quality from 1 (excellent) to 3 (nonevaluated). The Wilcoxon signed-rank test was used to analyze the image quality in both groups using a 16-segment coronary artery model.

## 3. Results

**Figure 3:**
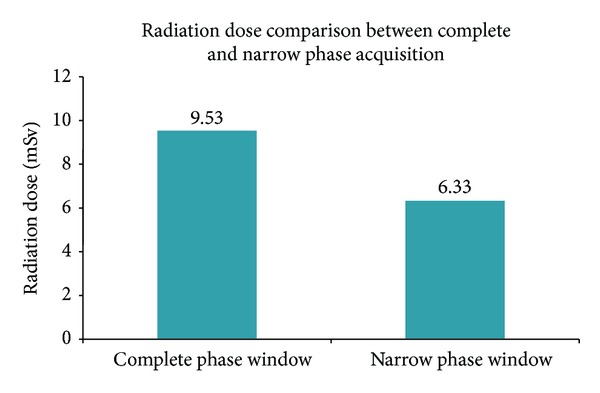
Radiation dose comparison between complete and narrow phase window acquisition at 320-row MDCTA.

Both groups were statistically comparable as no significant difference was found between them with respect to their heart rate (*P* = 0.59), BMI (*P* = 0.17), and imaging tube current mAs (*P* = 0.68). The median radiation dose (interquartile range, IQR) was significantly higher in those patients scanned during entire cardiac cycle R-R interval (Group B) versus this set of patients (Group A) imaged with prospectively defined 66–80% phase window (9.53 (6.70–12.62) versus 6.33 (5.3–8.66) mSv, *P* < 0.0001). Even after adjusting the patient's variables and other scanning parameters in analysis, results showed that patients imaged during entire cycle (Group B) were associated with higher radiation dose (33%, *P* < 0.001) Figure 3.

### 3.1. Image interpretability

Image quality assessed at the segmental level was better among those patients scanned at narrow phase window, that is, excellent (85%), good (14.87%), and nondiagnostic (0.08%) versus complete phase window, excellent (65%), good (33.14%), and non-diagnostic (1.39%); *P* < 0.0001 for differences (Table 3).

## 4. Discussion

Our study was aimed to determine the impact of different R-R window phase acquisition on radiation dose and image quality for 320-row MDCT in coronary artery evaluation. Our data adds to the prior study by Steigner et al. who using multiple phase windows demonstrated that 60–95% was most advantageous. In our use of 66–80% of the R-R interval, we have demonstrated good to excellent quality images correlated with decreased radiation exposure to the patients (33% reduction) with good image interpretability.

In the initial coronary MDCTA trials, excessive radiation exposure remained a major concern that limited wider acceptance of this imaging modality [6, 14, 15]. These concerns prompted the search for techniques to minimize radiation dose while maintaining image quality. Previous strategies have documented heart rate-independent methods that can be applied to minimize the radiation dose including anatomy-adapted tube-current modulation [16, 17] and reduction in tube voltage [18–20]. Similarly, there are several accepted heart rate-dependent methods that can be applied to reduce radiation dose: (a) decreasing tube current during the systolic phase of electrocardiogram (tube current modulation) [21], (b) decreasing tube current during the nonreconstructed phase [22], (c) using a rate-adaptive pitch [23], and (d) sequential ECG triggering [24]. All these methods are dependent and somewhat limited by the temporal resolution and the function of the CT technology.

Aquilion ONE is a cone beam MDCT with a 320-row 0.5 mm detector array. The 320-row mode, at present, functions primarily as a cardiac scanner, that is, volume scan mode. Coronary MDCTA with 320-row mode has sufficient craniocaudal coverage (160 mm) making it possible to image the entire heart in a single heartbeat and one gantry rotation. Using this approach, the patient is exposed for only 350 ms, thereby creating the possibility of reducing radiation exposure substantially when compared to the overlapping rotations employed with the traditional helical MDCTA [3, 14, 15, 25].

All patients in our study were scanned at maximum volume scan length (VSL) 160 mm (to make both groups comparable), but our previous study has shown a 33–46% reduction in radiation dose by decreasing VSL from 160 to 140 or 120 mm depending upon patient's heart length [25]. In this retrospective study, mean radiation dose noted for patients imaged using 320-row MDCT was 6.33 mSv, which is comparable to prospective gating on 64 MDCT scanners in patients with heart rates <65 bpm.

Currently, Complete phase window acquisition with tube modulation mode is also used for patients with irregular heart rates or rate variations during breathing exercise or the actual scan in addition to functional analysis. Sudden abrupt change in heart rate during the scan can result in some loss of valuable data and motion artifact. Although mean heart rate was the same in both groups, a higher or variable heart rate as described is the most likely reason for slightly better image quality in cases of narrow phase window versus complete phase window patients.

### 4.1. Limitation

There are limitations to our study as it was a single-center, retrospective analysis, with a small number of patients. Further prospective studies with a larger number of patients in multiple sites could better evaluate the effect of window phase selection on radiation dose and image quality related to 320 MDCT scanners. Also, the focus of the study was image quality and not diagnostic accuracy; however, adequate image quality is a prerequisite for the assessment of diagnostic accuracy. Finally, our study is subject to the bias of a single center; a multicenter study is needed to further validate the concept.

## 5. Conclusion

In conclusion, scan mode and imaging parameters are critical determinants of radiation exposure and image quality on 320-row MDCTA. The median radiation doses of MDCTA vary significantly between patients scanned with complete versus narrow phase window acquisition. Low radiation dose and comparable diagnostic imaging quality may suggest an increased use of narrow window selection of R-R interval in selected patients. Given the potential for low radiation dose with volume scanning, further prospective studies for evaluation of diagnostic accuracy, efficacy, and impact on patient outcomes would be advantageous.

## Figures and Tables

**Table 1:** Summary of patient characteristics for two groups undergoing imaging using complete versus narrow phase window acquisition at 320-row MDCTA.

Patient characteristics	Complete phase window	Narrow phase window
BMI	28 ± 5	29 ± 6
HR (bpm)	57 ± 7	58 ± 7
Age	63 ± 16	61 ± 13
Gender (male : female)	14 : 13	63 : 24

**Table 2:** Typical imaging and reconstruction parameters for complete versus narrow phase window acquisition at 320-row MDCTA.

	Complete phase window	Narrow phase window
Tube voltage	120 kVp	120 kVp
Tube current-time product	400 mA	400 mA
R-R phase	Complete phase with dose modulation; peak dose at 75%	66%–80%
Reconstruction filter kernel	Standard FC3	Standard FC3
Reconstruction field of view	≤250 mm	≤250 mm
Slice thickness: increment	0.5 mm : 0.3 mm	0.5 mm : 0.3 mm

**Table 3:** Summary of image quality for two groups undergoing imaging using complete versus narrow phase window acquisition at 320-row MDCTA.

Image Quality (i) Excellent (ii) Diagnostic (iii) Nondiagnostic	Narrow phase window (Group A) Segments (%)	Complete phase window (Group B) Segments (%)
	Total Patients = 87 Total segments = 1244	Total patients = 27 Total segments = 359
Excellent	1058 (85%)	235 (65%)
Diagnostic	185 (14.87%)	119 (33.14%)
Nondiagnostic	1 (0.08%)	05 (1.39%)
